# SegGen: An Unreal Engine 5 Pipeline for Generating Multimodal Semantic Segmentation Datasets

**DOI:** 10.3390/s25175569

**Published:** 2025-09-06

**Authors:** Justin McMillen, Yasin Yilmaz

**Affiliations:** Electrical Engineering Department, University of South Florida, Tampa, FL 33620, USA; jmcmillen@usf.edu

**Keywords:** computer vision, multimodal data fusion, synthetic data generation, semantic segmentation

## Abstract

Synthetic data has become an increasingly important tool for semantic segmentation, where collecting large-scale annotated datasets is often costly and impractical. Prior work has leveraged computer graphics and game engines to generate training data, but many pipelines remain limited to single modalities and constrained environments or require substantial manual setup. To address these limitations, we present a fully automated pipeline built within Unreal Engine 5 (UE5) that procedurally generates diverse, labeled environments and collects multimodal visual data for semantic segmentation tasks. Our system integrates UE5’s biome-based procedural generation framework with a spline-following drone actor capable of capturing both RGB and depth imagery, alongside pixel-perfect semantic segmentation labels. As a proof of concept, we generated a dataset consisting of 1169 samples across two visual modalities and seven semantic classes. The pipeline supports scalable expansion and rapid environment variation, enabling high-throughput synthetic data generation with minimal human intervention. To validate our approach, we trained benchmark computer vision segmentation models on the synthetic dataset and demonstrated their ability to learn meaningful semantic representations. This work highlights the potential of game-engine-based data generation to accelerate research in multimodal perception and provide reproducible, scalable benchmarks for future segmentation models.

## 1. Introduction

Semantic segmentation is a cornerstone task in computer vision [1], enabling pixel-level understanding that is critical for applications ranging from autonomous driving [2] to environmental monitoring [3]. Although significant progress has been made in multimodal segmentation for road scenes and urban driving environments [4,5,6,7,8], there is still a clear gap in datasets for domains outside of these areas. In particular, tasks such as remote sensing and landscape analysis in remote areas (e.g., forestry analysis) have been largely under-served due to the high cost and labor-intensive nature of manually annotating large-scale datasets [9].

Synthetic data generation has emerged as a promising alternative, offering scalable, controllable environments with ground-truth annotations [8,10,11]. However, most existing pipelines are tailored to road scenes, leaving limited tools for other natural domains.

In this work, we present SegGen (https://github.com/Secure-and-Intelligent-Systems-Lab/SegGen (accessed on 23 August 2025)), an automated Unreal Engine 5 (UE5) pipeline design for generating multimodal semantic segmentation datasets. SegGen leverages procedural biomes and a spline-driven drone actor to rapidly produce diverse environments and aligned RGB, depth, and label imagery. Our proof-of-concept dataset covers a 1 mi^2^ synthetic forest with multiple tree species and terrains, and we validate SegGen by benchmarking three segmentation models.

Our contributions are:

- A modular UE5 pipeline for procedural scene generation and automated dataset capture, with pixel-perfect ground-truth labels.
- A proof-of-concept multimodal dataset of a heterogeneous forest (1169 samples, 1920 × 1080 resolution).
- Baseline semantic segmentation results using UNet [12], SegFormer [13], CMNeXt [8], and FuseForm [14].

## 2. Related Work

The evolution of deep learning in computer vision is closely tied to the availability of large-scale annotated datasets. However, creating datasets with pixel-level annotations for semantic segmentation is expensive and labor-intensive. In response, many studies have explored synthetic data generation as an alternative to reduce or eliminate manual labeling. In this section, we review synthetic data generation, game engine-based dataset pipelines, procedural content generation, multimodal approaches, and domain adaptation, as they apply to semantic segmentation tasks outside of autonomous driving.

### 2.1. Synthetic Data for Computer Vision

Early works demonstrated that computer-generated imagery can provide usable ground truth for vision tasks. Playing for Data [15] showed that video game environments can provide usable ground truth for tasks such as semantic segmentation and object detection. Subsequent datasets such as Virtual KITTI 1/2 [16,17], SYNTHIA [10], and surveys [6] harnessed simulated driving scenarios to create detailed pixel-level annotations for autonomous driving applications. More recently, Clabaut et al. [18] introduced a synthetic data approach for Sentinel-2 imagery segmentation, reducing the reliance on manual annotations in remote sensing. Borkman et al. [19] developed Unity Perception, which provides tools for generating synthetic datasets with perfect annotations for common vision tasks, and demonstrated models trained with synthetic data can outperform real-only baselines.. Complementing these efforts, Tobin et al. [20] and Nikolenko et al. [21] further highlighted strategies to narrow the gap between simulation and reality, a challenge that still persists in many synthetic data applications. Representative datasets are summarized in Table 1.

A persistent challenge across these efforts is bridging the domain gap, mitigated with domain randomization, GAN-based realism enhancement, or blending synthetic and real data [22].

### 2.2. Procedural Content Generation and Multimodal Approaches

Procedural content generation (PCG) creates diverse environments without extensive manual input [23]. Modern PCG frameworks available in recent versions of Unreal Engine 5 enable the rapid synthesis of complex scenes and realistic variations in environmental factors, such as lighting and geometry. These methods have been predominantly applied to urban and driving scenarios [24]. Recent work, such as Nunes et al. [25], highlights PCG’s potential to create forest environments specifically designed for training ML algorithms.

Multimodal datasets combining RGB, depth, and LiDAR provide richer representations. FinnWoodlands [26] integrates multiple modalities (RGB, depth, point clouds) to provide richer representations of forestry environments, paving the way for more robust semantic segmentation models. Lu et al.’s M2fNet [9] used procedural generation of a photorealistic virtual forest for tree detection and segmentation. Similarly, Sen12MS [27] leverages multispectral imagery for land cover analysis. SCaRL [11] and SDAC dataset [28] provide synchronized multimodal data from game engines, supporting safety-critical driving tasks. Recent work also extends multimodal synthesis to vision-language learning, with structured rendering for instruction-tuned datasets [29]. Representative procedural and multimodal dataset contributions are summarized in Table 2.

### 2.3. Domain Adaption and Beyond Autonomous Driving

Despite the advancements in synthetic dataset generation, the majority of existing research has been focused on domains such as autonomous driving, where synthetic data pipelines are well established. For remote sensing and forest monitoring, domain-specific datasets remain limited. Wang et al. proposed SegForest [30], a segmentation network designed for forest remote sensing images. In parallel, challenges such as those presented in the DeepGlobe [31] and Loveda [32] datasets show the critical need for domain-adapted approaches. Domain adaption techniques, as described by Tobin et al. [20] and Mullick et al. [33], are pivotal in transferring knowledge from synthetic data to real-world scenarios, increasing the generalizability of segmentation models in under-served contexts.

Recently, the SPREAD dataset [34] leveraged UE5 to generate high-fidelity synthetic forests for segmentation and tree parameter estimation. While SPREAD significantly advances synthetic data realism and diversity, our SegGen pipeline differs by offering a more generalizable and automated framework specifically focused on multimodal semantic segmentation across diverse remote sensing scenarios. SegGen introduces procedural biome-based generation for any conceivable biome type, automated drone navigation, and multimodal data collection capabilities, which enables rapid dataset customization and expansion to various environmental contexts beyond forestry alone. A direct comparison between SPREAD and our SegGen pipeline is provided in Table 3.

### 2.4. Integration and Gaps in Current Research

The literature reveals a trajectory from early game-based datasets to procedural UE5 pipelines and multimodal fusion. Yet, synthetic multimodal datasets for remote sensing remain scarce. Existing datasets are urban-centric, while remote sensing tasks require tailored modalities.

By integrating synthetic data generation [21], PCG [23,24,25], and domain adaptation [33], our SegGen pipeline provides scalable dataset generation for multimodal segmentation, broadening opportunities in underexplored remote sensing domains.

## 3. Methodology

**Figure 1:**
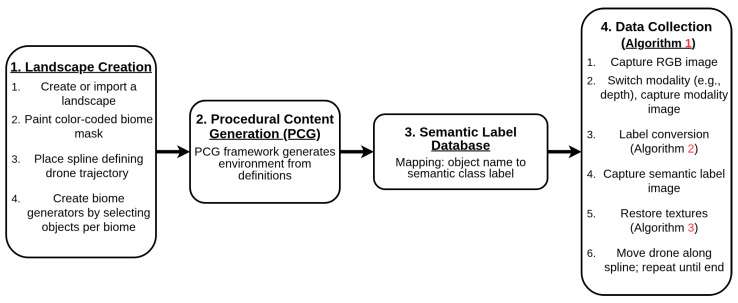
Overview of the complete pipeline for automated multimodal semantic segmentation dataset generation. The process flows from initial terrain generation through biome definition, semantic labeling, drone trajectory setup, and ends with automated multimodal data collection.

**Figure 2:**
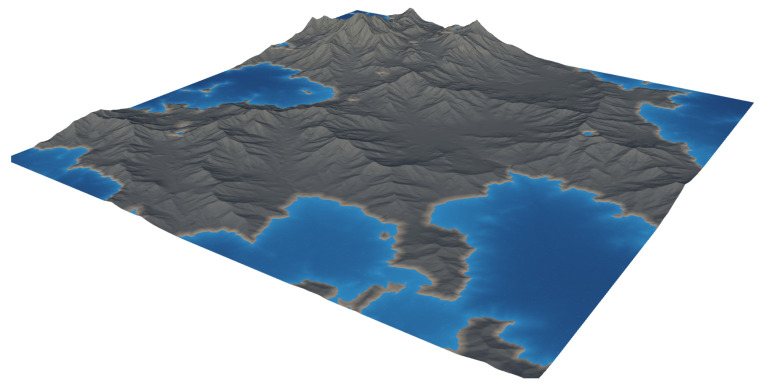
A landscape generated in Gaea. Using this software, users can create highly realistic natural environments for many different landscape types (e.g., mountainous, forested, swampy, desert, etc.).

**Figure 3:**
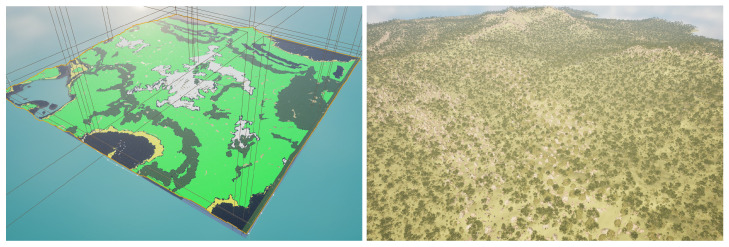
Birds-eye-views: (**Left**) Landscape after overlaying the biome-mask. (**Right**) Landscape after applying Biome-based PCG.

**Figure 4:**
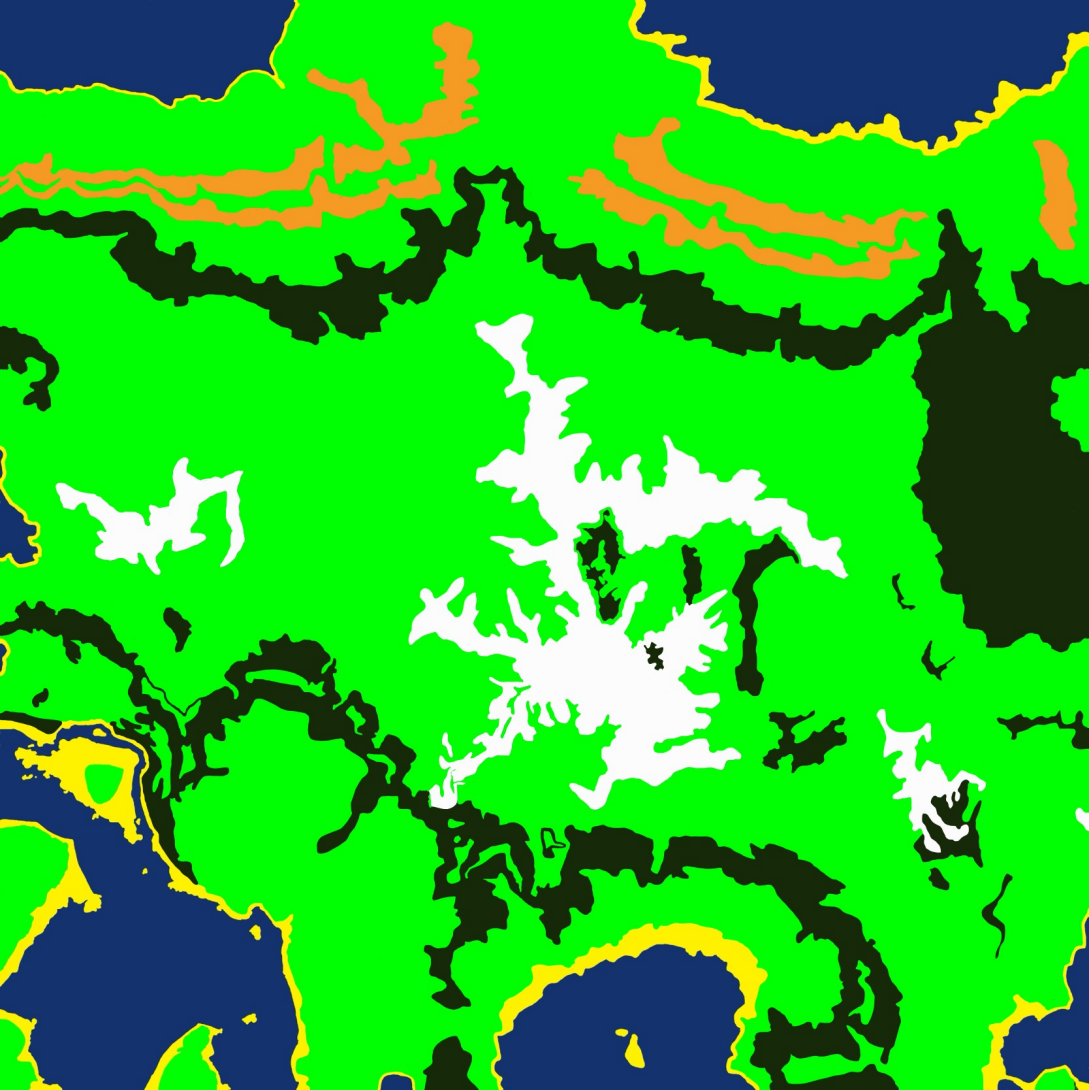
The biome mask overlay which informs the procedural biome generator where to place specific biomes. Different colors represent different biomes.

Our proposed pipeline for generating multimodal semantic segmentation datasets, which is shown in Figure 1, consists of several sequential stages designed for simplicity and flexibility. First, we create or import a suitable virtual landscape. In this proof-of-concept demonstration, we utilize Gaea (https://quadspinner.com (accessed on 23 August 2025)), a freely available physics-based terrain generation tool, to produce a realistic one-square-mile mountainous environment, which we then import into UE5. A biome created in Gaea is shown visually in Figure 2. Next, we define distinct biome regions by painting a color-coded biome mask directly onto the imported landscape, as illustrated in Figure 3. Each color corresponds to a specific biome type, guiding UE5’s procedural biome generator to populate these regions accordingly. The completed biome mask is shown in Figure 4. After defining biome regions, we configure the biome definitions by selecting static meshes and adjusting parameters such as object spawn density, placement rules, and spacing constraints. Following biome configuration, we set up the semantic label database, as described in Section 3.1, which maps object names to their corresponding semantic class labels. Once the environment and labels are prepared, we define a spline path within the environment for our drone actor to follow, as in Section 3.2. Finally, the dataset is generated by running our automated multimodal data collection process, detailed further in Section 3.2 and Section 3.4, as well as Algorithm 1. This workflow enables efficient creation and customization of synthetic multimodal datasets tailored to diverse research scenarios. A high-level step-by-step guide is detailed further in Section 3.5, and a more technical version is available on GitHub (https://github.com/Secure-and-Intelligent-Systems-Lab/SegGen (accessed on 23 August 2025)).
**Algorithm 1** Spline-Based Multimodal Data Collection**Require:** Spline path *S* defined by user, Drone Actor *D*, Modalities *M* =      {RGB,Depth,…}, Camera parameters (f,a)**Ensure:** Multimodal dataset along spline path *S*  1: Initialize Drone Actor *D* at the start of spline *S*  2: Set camera parameters (focal length *f*, aperture *a*)  3: **while** Drone *D* has not reached the end of spline *S* **do**  4:     **for** each modality m∈M **do**  5:         **if** *m* is RGB **then**  6:             Capture RGB image using virtual camera  7:             Store RGB image in RGB folder  8:         **else**  9:             Activate Modality Switcher for modality *m*10:             Capture modality image (e.g., depth map)11:             Store image in corresponding modality folder12:         **end if**13:     **end for**14:     Activate Semantic Label View15:     Capture and store semantic label image in labels folder16:     Move Drone *D* to next predetermined sampling location along spline *S*17: **end while**

### 3.1. User-Configurable Object–Class Mapping

In UE5, every object in the game world, be it a tree, rock, building, pebble, etc., has an object name. By sorting and filtering these objects by their names, we can then assign them different materials. Using this technique, we assign each object to a semantic class by ‘painting’ the object a single color.

To drive both this label texture conversion and original texture restoration, we maintain two core data structures:

- Database
  DB:{substring↦LabelMaterial}
  – Populated by the user prior to runtime.
  – Each key is a name fragment (e.g., "alder" to refer to all types of alder trees), and each value is the corresponding semantic label material.
- Texture Store
  T:{ObjectID↦OriginalTexture}
  – Filled during label conversion to remember each object’s pre-label material.
  – Consumed during restoration to reapply the original materials.

Workflow:

1. In Algorithm 2 (Label Conversion):
  - For each nearby object *O*, if O.ID contains a key κ in DB, then
    T[O]←O.texture,O.texture←DB[κ].
2. In Algorithm 3 (Texture Restoration):
  - For each modified object *O*, retrieve t=T[O] and set
    O.texture←t.
  - Clear *T* to prepare for the next capture cycle.

**Figure 5:**
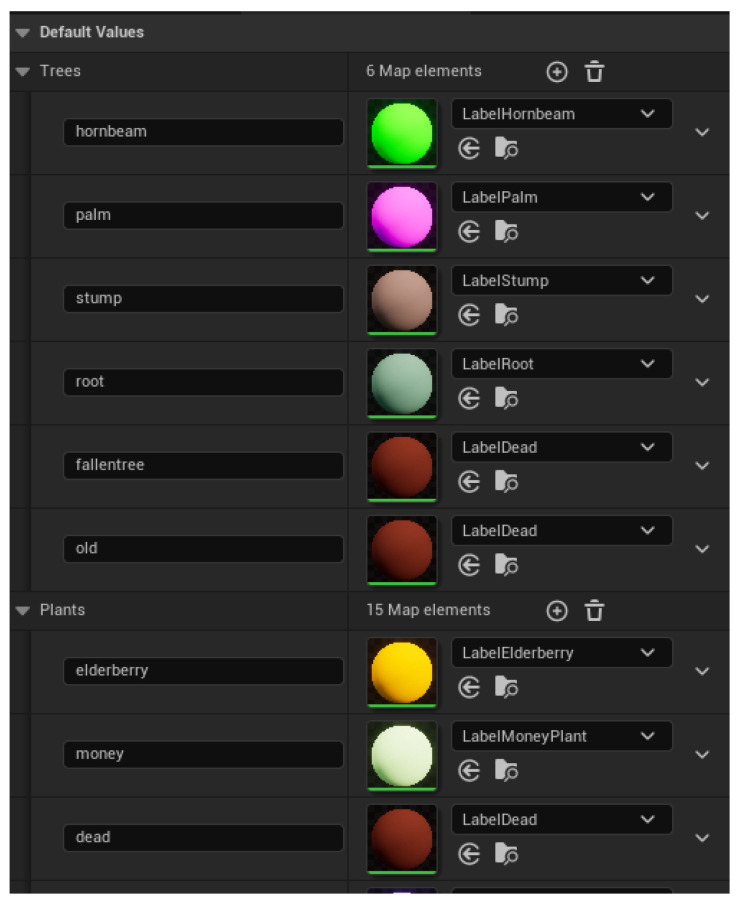
The database where substrings (on the **left**) and their semantic-class mappings (on the **right**) are stored. These are created by the user prior to collecting data.

By keeping the semantic-class mapping (DB) separate from the original-texture store (*T*), the semantic labeling pipeline is extremely flexible and scalable. Adding a new semantic class is as simple as inserting one more substring→*LabelMaterial* pair in DB, with no changes needed to the core algorithms. An example semantic class mapping database in UE5 is shown in Figure 5.
**Algorithm 2** Semantic Label Conversion Logic**Require:** Drone Position *P*, Horizontal Radius *r*, Object-Class Mapping Database DB  1: Identify all landscape streaming actors within radius *r* of position *P*  2: **for** each identified landscape streaming actor *A* **do**  3:     **for** each object *O* in landscape streaming actor *A* **do**  4:         **for** each substring *S* in DB **do**  5:             **if** object name contains substring *S* **then**  6:                 Store original texture of object *O*  7:                 Replace object *O*’s texture with semantic label material from DB  8:                 Break for loop  9:             **end if**10:         **end for**11:     **end for**12: **end for**

**Algorithm 3** Texture Restoration Procedure
**Require:** Modified Object Set *O*, Stored Original Textures *T*  1: **for** each object *o* in *O* **do**  2:     **if** object name *o* exists in stored textures *T* **then**  3:         Retrieve original texture $t_{o}$ from stored textures *T*  4:         Apply original texture $t_{o}$ back to object *o*  5:     **end if**  6: **end for**  7: Clear stored original textures *T* for next iteration


### 3.2. Drone Platform Spline Actor

**Figure 6:**
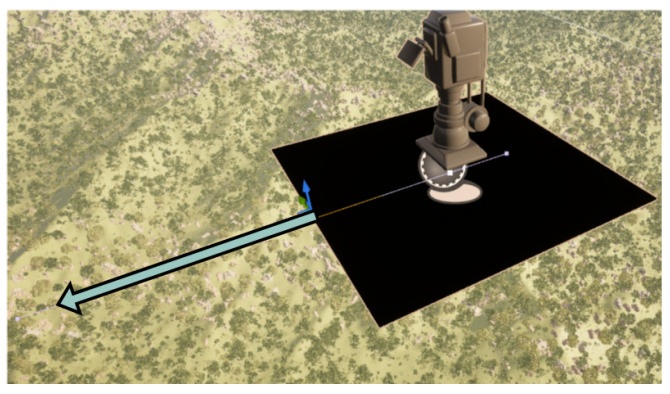
The drone actor which collects multimodal data. In this image, the depth modality cube is visible. The arrow indicates the direction of the spline which the drone will follow.

We implemented a drone actor capable of following a user-defined spline path within the UE5 environment. A closeup of the drone actor is shown in Figure 6. The user manually places the spline to define the desired data collection trajectory. For remote sensing purposes, this simulates a drone; however, this can be adapted to follow the behavior of any vehicle. At each data collection point along the spline (defined in the drone logic), the drone executes the logic presented in Algorithm 1, which is described at a high level by the following procedure:

1. RGB Capture: The drone first captures an RGB image using the onboard virtual camera configured with specific aperture and focal length settings (user-defined specifications).
2. Modality Switching and Capture: After the RGB image is stored, the drone activates the modality switcher, capturing images from additional sensor modalities (e.g., depth). Each modality is activated sequentially, and its respective image is captured and stored.
3. Semantic Label Capture: Finally, the drone captures a semantic segmentation label image.

Each captured image is stored separately in modality-specific directories. After completion at one point, the drone moves a user-specified incremental distance along the spline and repeats the data collection process, ensuring consistent sampling intervals.

### 3.3. Modality Switcher

**Figure 7:**
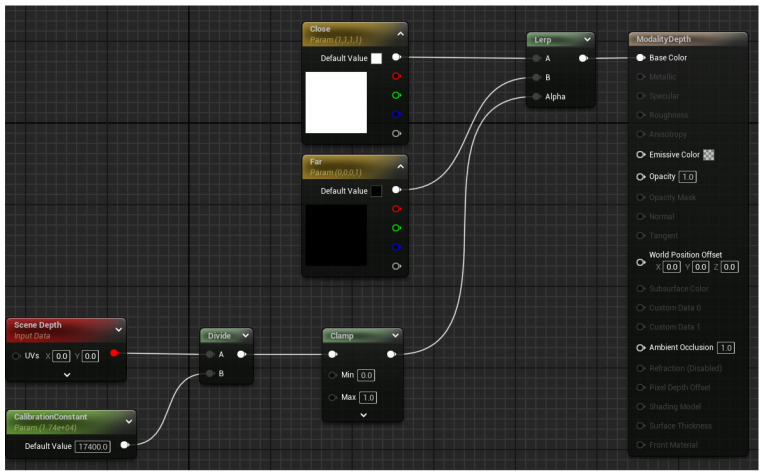
UE5 blueprint view of the depth modality material.

To enable rapid switching between different sensor modalities, we employ a modality-switching mechanism utilizing a flat cube geometry positioned directly in front of the drone’s viewport. This cube is textured dynamically based on the active modality:

For this proof-of-concept implementation, we demonstrate using a depth modality as an additional data acquisition sensor. The material blueprint dynamically encodes depth values into grayscale intensities, providing a depth map. The UE5 Blueprint is shown in Figure 7, where scene depth (red block), $d_{p}$, is captured through the material for each pixel. The depth value $d_{p}$ is normalized by dividing by a calibration constant $d_{cal}$ and clamped to produce an intensity value $I_{p}$ between 0 and 1, calculated as follows:

(1) $$I_{p}=\mathrm{clamp}\left(1-\frac{d_{p}}{d_{cal}},\;0,\;1\right)$$

This ensures that pixels at zero distance ($d_{p}=0$) are rendered white (intensity of 1), and pixels with distances equal to or greater than $d_{cal}$ ($d_{p}\geq d_{cal}$) are rendered black (intensity of 0). This approach can be easily adapted to other modalities such as roughness, specularity, albedo, etc., through the use of built-in modules for UE5, in place of the scene depth block.

### 3.4. Label Conversion Logic

To generate semantic segmentation labels accurately, we implement logic to swap scene textures with predefined label materials:

1. Spatial Filtering: First, the system identifies all landscape streaming actors within a 100 m horizontal radius of the drone’s current position, excluding distant actors to improve computational efficiency.
2. Material Replacement: For each identified actor, we iterate through contained objects. Objects whose names contain specific user-defined substrings are recognized and assigned corresponding semantic label materials.
3. Texture Restoration: Original textures are stored temporarily, enabling reversion to the original textures after the semantic label image has been captured.

Algorithm 2 provides a detailed summary of this label conversion logic, while Algorithm 3 covers the reverse case:

By integrating these four components into a cohesive workflow, our pipeline generates extensive, accurately labeled multimodal datasets suitable for training semantic segmentation models in varied domain-specific scenarios.

### 3.5. Creating New Datasets

The creation of new datasets within our SegGen pipeline involves several straightforward steps, leveraging procedural generation, user-defined settings, and automated data collection. It is recommended that the user reference the SegGen GitHub repository (https://github.com/Secure-and-Intelligent-Systems-Lab/SegGen (accessed on 23 August 2025)) for example files and default configurations, as well as a detailed walkthrough. The step-by-step workflow is described below:

Step 1: Environment Setup

The initial step is environment setup, which includes terrain generation and biome definition. Terrain can be procedurally generated using tools like Gaea, or created manually using the landscape sculpting tools available inside UE5. After importing/creating the terrain, users define distinct biome regions using a biome mask (Figure 4) that instructs UE5’s procedural generation system on how to populate these regions. The biome mask can be created in any image editor, such as MS Paint or GIMP. Each color in the image will need to mapped to a biome color inside UE5, accessible through each biome definition’s settings. Each biome the user wishes to generate, such as forests, rocks, or grasslands, can be customized by adjusting parameters such as density, distribution patterns, and asset selection, accessible through the biome generator’s settings. By default, the SegGen GitHub repository contains biome generators and definitions adapted for most use cases for natural scenery. The objects which are spawned by the generators are defined in the biome’s definition files, also available in the SegGen GitHub repository.

Step 2: Semantic Class Mapping

Users must define an object-to-class mapping, linking specific semantic labels to corresponding scene objects. This mapping is user-configurable and maintained within a database (DB), associating object names or substrings (e.g., “oak”) to specific semantic label materials. Users update this DB prior to dataset creation. For reference, we provide two example class mappings. One with consolidated labels, as used in our use-case dataset in Section 4, and a diverse class mapping (Figure 5), which contains mappings for a wide variety of classes (50+ classes of trees, plants, rocks).

Step 3: Drone Trajectory Planning (Optional)

The dataset generation pipeline will work either on foot or from a drone point of view. If using the player-character actor as the data collection source, only sample collection is currently supported (the user must press the collect sample button each time and manually position the player-character where they want to collect the sample). If using a drone, defining the trajectory for data collection is required. The drone actor trajectory is specified using the drone’s spline actor within the UE5 environment. Users define the spline path, and can set the desired spacing between collected samples, accessible through the drone’s settings. The spline actor then follows this user-defined trajectory, allowing for reproducible data collection.

Step 4: Automated Data Collection (if using a drone)

The data collection phase is fully automated and follows the procedure outlined in Section 3.2 and Algorithm 1. Users simply need to press the ‘collect data’ keybinding on the drone actor.

Step 5: Data Post-processing (Optional)

Label images are collected in RGB format. For use in downstream tasks, they must usually be converted to integer label format. To accomplish this, an RGB → Semantic class mapping is required. One method to accomplish this is to use a K-Means algorithm with $k=N_{classes}$ clusters. We anchor the K-Means clusters to a label material color defined by the user. The pixels are then clustered and assigned to their respective classes based on the minimum distance to a cluster center. This method ensures that each pixel is mapped correctly, and removes and chance at mislabeling or skipping over pixels which may slightly differ in color due to rendering artifacts, noise, anti-aliasing, or any other aberrations which may slightly alter a pixel’s color. In the SegGen GitHub repository we provide a standalone python file which can perform this mapping step.

By following these steps, users can efficiently create tailored, large-scale multimodal datasets suited for diverse semantic segmentation tasks, significantly accelerating research and development in specialized application domains.

### 3.6. Adding New Data Collection Modalities

A key design principle of SegGen is modularity, allowing researchers to generate datasets across multiple sensing domains. Unreal Engine 5 (UE5) natively supports a variety of rendering passes beyond RGB, such as albedo, surface normals, and roughness. These can be implemented exactly like the depth data collection modality, enabling the creation of multimodal datasets without much additional configuration.

For LiDAR data, a sensor plugin has been developed and is publicly available on GitHub (https://github.com/metabotics-ai/MetaLidar?tab=readme-ov-file (accessed on 23 August 2025)). This plugin integrates with UE5 to provide physically realistic point cloud simulations, including both distance and return intensity values. The latter is based on object material properties, such as surface color and reflectivity, thereby approximating real-world LiDAR behavior. The generated point clouds can be used for tasks such as 3D semantic segmentation, object detection, or scene reconstruction.

While visual data generation is supported natively within UE5, creating non-visual modalities such as multispectral or SAR data requires additional configuration. For multispectral imagery, one approach is to apply custom material filters or shaders that isolate narrow wavelength bands (e.g., near-infrared, shortwave infrared) and render separate passes corresponding to these channels. Similarly, synthetic SAR can be approximated by simulating radar backscatter, either through custom shaders that model reflectivity as a function of surface roughness and angle or by using physics-based plugins that emulate radar wave propagation. Implementing these modalities, while feasible, requires more extensive pipeline development and careful calibration to achieve physical realism. A thorough exploration of non-visual modality generation is beyond the scope of this work but represents a valuable direction for future research.

## 4. Use Case: Fine-Grained Landscape Classification

This section documents the experimental protocol used to benchmark the proposed ue5-forest pipeline on an automatically labeled, multimodal dataset. We first describe the dataset and its statistics, then detail the model selection, training protocol, and evaluation criteria.

### 4.1. Dataset

**Figure 8:**
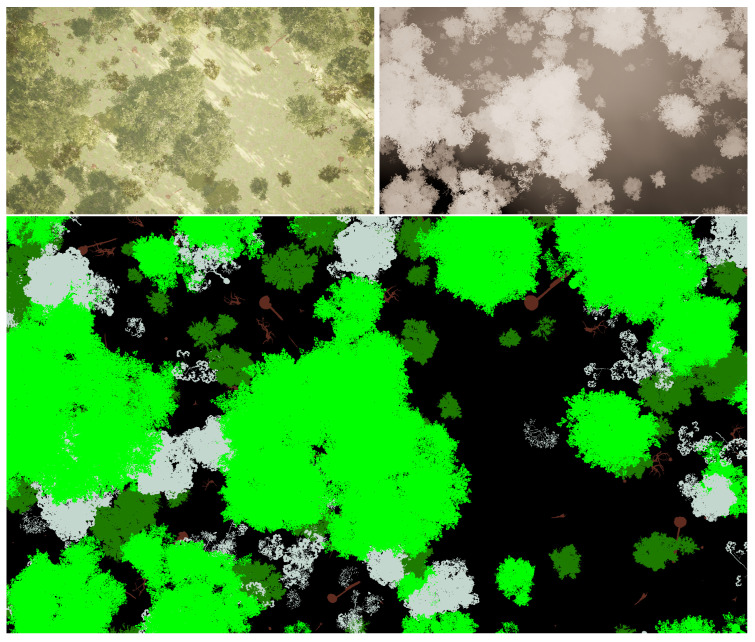
Sample from the ue5-forest dataset. The depth (**top right**) and label (**bottom**) images are perfectly aligned with the RGB frame (**top left**). The label image shows class-specific annotations before morphological smoothing to showcase the label fidelity (see Section 4.1). Different colors in the label image represent different semantic classes.

We procedurally generated a $1\times 1\;{\mathrm{mi}}^{2}$ mountainous landscape in Unreal Engine 5 and populated it with six semantic categories—Ground, European Beech, Rocks, Norway Maple, Dead Wood, and Black Alder, as detailed in Table 4. A virtual drone followed a user-defined spline that adequately covers the scene from a roughly fixed altitude of 25 m. RGB, depth, and label frames were captured with a vertical overlap of approximately 20% (10% top, 10% bottom), yielding 1169 image pairs at a native resolution of 1920×1080. A sample from the generated dataset is presented in Figure 8.

**Figure 9:**
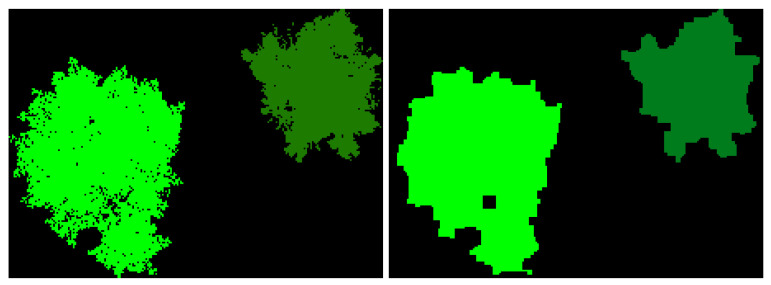
Before and after label postprocessing. (**Left**) Pre-postprocessing, the image contains extremely high-resolution ground truth labels, which is too granular for our task. (**Right**) After postprocessing, excess high-frequency data is removed which allows the model to learn the different foliage representations better.

Pixel-perfect ground-truth masks were generated using the label creation pipeline described in Section 3.4. A laptop consisting of a 4090 Mobile GPU and 32 GB of RAM was used to generate the dataset; however, any computer able to load the desired UE5 map is able to collect data. In other words, the SegGen framework does not have any computing requirements above the UE5 engine itself. The time to collect the 1169 image pairs was approximately 4 h. Actual time to collect a dataset depends on two main factors, namely number of images and number of modalities. The majority of time in between sample collections is buffer to allow the camera aperture and lighting to adjust between modality switching. These can be fine-tuned by the user to increase collection speed based on the user’s needs and configuration. The class-specific colored label images were encoded into integer labels using a python 3.11 script which maps each pixel to a semantic class based on its color. The resulting integer label images contain extreme high-frequency information, so they were smoothed using the following two-stage morphological filter implemented in OpenCV: a 3×3 opening (erosion → dilation) that removes one-pixel noise, immediately followed by a 5×5 closing (dilation → erosion) that seals pin-holes and smooths ragged boundaries. This open–close sequence preserves object topology while suppressing the extreme high-frequency artifacts introduced by per-pixel color encoding. An example of the before and after postprocessing is shown in Figure 9. The dataset is then shuffled and split 80:20 into training and test sets.

Table 4 reports the class-wise pixel distribution over the entire training split. The canopy classes together dominate the label space, while Dead Wood and Black Alder are distinctly under-represented, making the task class-imbalanced and ecologically realistic.

To emulate realistic aerial-imaging noise and to combat over-fitting, each input pair undergoes:

- Random horizontal flip (p=0.5),
- Random horizontal rotation $\left[-75^{\circ },75^{\circ }\right]$,
- Random scale and crop to 768×1280 with scale factor [0.8,1.2],
- Gaussian blur with σ∈[0,1].

### 4.2. Model Selection

We compare five architectures spanning the design spectrum from convolutional to Transformer-based models. UNet [12] is a classical CNN with an encoder–decoder structure and skip connections, enabling the integration of low- and high-level features for precise segmentation. It is widely adopted for its simplicity and strong performance across segmentation tasks.

SegFormer [13] replaces convolutions with a MiT-B4 Transformer encoder that captures multiscale contextual information via attention. A lightweight MLP decoder then reconstructs the segmentation output, offering high accuracy with efficient computation.

CMNeXt [8] extends the CMX framework with architectural refinements that improve both accuracy and efficiency. It employs a simplified cross-modal fusion module, optimized backbone design, and reduced computational complexity, enabling real-time multimodal segmentation with competitive performance across diverse benchmarks.

FuseForm [14] adopts a hybrid Transformer-based design tailored for multimodal segmentation. It employs modality-specific MiT encoders (B4 for RGB and B2 for depth), a shared Transformer decoder, and cross-modal attention fusion blocks that dynamically integrate information across modalities for more informed predictions.

Each model is evaluated in two variants: (1) using RGB only, and (2) using RGB+Depth. For UNet and SegFormer, the input layer is extended to accept four channels. For CMNeXt and FuseForm, multimodal data fusion is handled their their respective fusion modules.

### 4.3. Training Protocol

All models are trained with similar hyper-parameters to ensure a more fair comparison:

- Input size: 1280×768 during training; at inference we use a resolution of 1792×1024.
- Optimizer: AdamW ($\beta_{1}\;=\;0.9,\;\beta_{2}\;=\;0.999,\;w_{d}\;=\;10^{-2}$).
- Epochs: 100 epochs for UNet, SegFormer, and FuseForm, 300 epochs for CMNeXt.
- Learning rate: $6\times 10^{-5}$ with a 10-epoch linear warm-up (10% of base LR), followed by polynomial decay (p=0.9).
- Batch size: 2 image pairs.
- Loss: pixel-wise cross-entropy.

We adopt two standard metrics for evaluating dense prediction tasks: Intersection-over-Union (IoU) and Overall Accuracy (OA), along with Per-Class Accuracy (CA) for detailed class-wise behavior.

Intersection-over-Union (IoU).

For class *c*,

$${\mathrm{IoU}}_{c}=\frac{|P_{c}\cap G_{c}|}{|P_{c}\cup G_{c}|},$$

where $P_{c}$ is the set of predicted pixels for class *c* and $G_{c}$ is the set of ground-truth pixels for class *c*. The Mean IoU (mIoU) is the unweighted average over all *C* classes:

$$\mathrm{mIoU}=\frac{1}{C}\sum_{c=1}^{C}{\mathrm{IoU}}_{c}.$$

Per-Class Accuracy (CA).

Per-class accuracy for class *c* is defined as the proportion of correctly predicted pixels among the total ground-truth pixels for that class:

$${\mathrm{Acc}}_{c}=\frac{|P_{c}\cap G_{c}|}{|G_{c}|}.$$

This highlights how well each class is individually recognized, particularly important in imbalanced settings.

Overall Accuracy (OA).

Overall accuracy measures the percentage of all pixels that are correctly classified:

$$\mathrm{OA}=\frac{\sum_{c=1}^{C}\left|P_{c}\cap G_{c}\right|}{\sum_{c=1}^{C}\left|G_{c}\right|}.$$

Unlike mIoU, OA is dominated by high-frequency classes and may not reflect performance on rare categories.

In class-imbalanced contexts such as ecological mapping, mIoU is the preferred headline metric, as it gives equal importance to each semantic class regardless of its frequency. OA and CA are reported as supplementary diagnostics.

## 5. Results

This section assesses the effectiveness of the proposed SegGen pipeline through a controlled comparison of three different computer vision architectures. All experiments are conducted on the synthetic dataset introduced in Section 4, which contains 1169 paired RGB–depth image–label tuples spanning six semantic classes (Ground, Beech, Rocks, Maple, Dead, Black Alder).

Each model is trained twice: (i) with RGB only and (ii) with RGB-depth input, using identical optimization settings to isolate the effect of the additional modality. Performance is reported in terms of mean Intersection-over-Union (mIoU) and Overall Accuracy (OA) as well as per-class IoU and accuracy to highlight behavior on minority categories.

Table 5 and Table 6 summarize the quantitative results, while Section 5.1 provides an in-depth discussion of aggregate trends, class-specific phenomena, and the implications for multimodal fusion in environmental monitoring.

### 5.1. Analysis

This synthetic benchmark exhibits a challenging class distribution, with visually dominant categories such as *Ground*, *Beech*, and *Rocks* accounting for the bulk of the pixels, while minority classes (*Dead*, *Alder*) occupy only a small fraction. Table 5 and Table 6 report mean Intersection-over-Union (mIoU) and Overall Accuracy (OA). To make the cross-model trends explicit, we summarize the aggregate scores and the effect of introducing depth in Table 7. All values are percentages; Δ denotes the change (RGB → RGB–Depth) in percentage points.

FuseForm outperforms the other methods on both metrics, confirming that its transformer–CNN hybrid decoder and multimodal cross attention for modality fusion leverages global–local context more effectively than purely convolutional (UNet) or purely transformer-based (SegFormer) designs. Across models, depth does not improve performance; the mean drop is 1.45 pp for mIoU and 0.33 pp for OA. FuseForm and CMNeXt suffer the least severe performance regression, showcasing their more advanced fusion methods.

Inspection of Table 5 and Table 6 reveals that high-frequency classes (*Ground*, *Beech*, *Rocks*) remain virtually unchanged when depth is added, whereas rare, geometrically intricate categories (*Dead*, *Alder*) suffer the largest declines (≈3 pp mIoU for FuseForm). We discuss the reasoning behind this in Section 5.2.

OA is inflated by the prevalence of easy background pixels and therefore masks the depth-induced confusion in the minority classes. mIoU, which weights each class equally, exposes these errors and is the headline figure for ecological monitoring tasks that rely on reliable detection of scarce phenomena (e.g., deadwood for fire-risk assessment).

### 5.2. Multimodal Performance Considerations and Limitations of Depth Fusion

While depth is often useful in multimodal perception, for species-level segmentation of vertically overlapping canopies, depth can be misleading or actively harmful if fused naively with RGB. In the benchmark dataset, every tree species—including *Beech*, *Maple*, *Alder*, and *Dead*—occupies the full vertical span of the canopy. As a result, the depth modality exhibits high intra-class variance and low inter-class separability: different species often occupy similar depths at a given (x,y) location, while a single species can appear across the entire depth range from trunk to crown. Therefore, depth provides little discriminative information for species-level segmentation.

When this depth signal is fused directly with RGB in an early-fusion pipeline, the network must reconcile two modalities with conflicting inductive biases. RGB encodes fine-grained texture differences between species, while depth implicitly encourages clustering based on vertical geometry. This mismatch can result in *gradient conflict*, where optimization steps favored by one modality degrade the performance of the other. Table 8 summarizes three failure modes specific to this setting: semantic non-discriminativeness, gradient conflict, and capacity dilution. These failure modes show the intrinsic limitations of early depth fusion; rather than assisting classification, depth can confuse the network.

Moreover, Table 7 (Section 5.1) shows that adding depth consistently decreases mean IoU across all models. The largest drops occur for the underrepresented classes *Dead* and *Alder*, which already suffer from texture ambiguity. These declines indicate that depth actively hurts fine-grain classification under naive fusion strategies.

#### On the Possibility of Sensor Misalignment

Even though the dataset is synthetically rendered, minor misalignments between RGB, depth, and label frames may still arise. One plausible source is procedural foliage animation during inference. Unreal Engine 5 enables subtle physical effects such as wind gusts, which may cause slight shifts in leaf and branch positions between the RGB capture, depth capture, and label rendering passes—especially when captured at intervals. While visually imperceptible, such pixel-level displacement can break pixel-perfect modality alignment and introduce noise at object boundaries. This would disproportionately affect thin or sparsely connected structures like deadwood and fine branches, further compounding performance loss in those classes.

### 5.3. Mitigation Strategies

To address the limitations identified above, we propose the following strategies:

- Late or selective fusion: Introduce depth at coarse resolutions or modulate its contribution via learned attention, rather than concatenating raw features early.
- Modality-aware loss weighting: Downscale depth loss contributions relative to RGB for classes known to be depth-insensitive.
- Ablation sanity check: Train a depth-only model; if mIoU approaches random or background-only accuracy, it confirms that depth is non-discriminative for this task.
- Optical alignment test: Overlay label boundaries on both RGB and depth to visually inspect subpixel displacement. If present, consider re-rendering with animation disabled.

These findings underscore that multimodal fusion is not universally beneficial. In tasks like species-level forest segmentation, where class identity is decoupled from 3D structure, the inclusion of geometric modalities without proper handling may degrade performance—despite perfect synthetic alignment. Future work should explore modality-aware fusion schemes, including confidence-based gating and adaptive attention, to filter out modality noise when signal content is uninformative or noisy.

### 5.4. Synthetic-to-Real

While the datasets generated by SegGen are synthetic, they provide a valuable platform for studying synthetic-to-real transfer in semantic segmentation and other computer vision tasks. Models trained on SegGen-generated imagery could be adapted to real-world data using established domain adaptation techniques, such as fine-tuning with a small set of real images, adversarial domain alignment, or style-transfer methods to reduce domain gap. Additionally, SegGen’s modular framework allows the introduction of photorealistic assets, diverse environmental conditions, and variable scene layouts, all of which can help narrow the synthetic-to-real gap. The UE5 asset store contains myriad different 3D-scanned assets which are scanned by professionals, meaning the asset quality is as close to realistic as possible. Although exploring these methods is beyond the scope of this work, SegGen provides a strong foundation for future investigations into synthetic-to-real transfer and the development of models that generalize effectively.

## 6. Conclusions

In this work, we introduced a fully automated Unreal Engine 5 pipeline, SegGen, for generating multimodal semantic segmentation datasets via procedural biome generation and a spline-driven drone actor, achieving high-throughput capture of aligned RGB and other modalities, alongside pixel-perfect label images. The proof-of-concept forest dataset, comprising 1169 image tuples at 1920 × 1080 resolution across six semantic classes, demonstrates the pipeline’s scalability and flexibility for domain-specific scenarios. Our framework can be leveraged for real-world tasks including automated forest monitoring, remote sensing-based biodiversity assessment, and training or evaluation of multimodal perception systems for environmental robotics.

Through a controlled benchmarking involving UNet, SegFormer, and hybrid FuseForm architectures, we showed that FuseForm attains the highest mean Intersection-over-Union (mIoU) of 85.83% on RGB inputs and exhibits the least performance degradation when incorporating depth, showing the importance of solutions designed from the ground up to work with multimodal data. Importantly, our analysis revealed that naively fusing a non-informative depth modality can harm fine-grain classification (particularly for underrepresented classes) due to semantic non-discriminativeness, gradient conflict, and capacity dilution.

To address these multimodal challenges, we recommend selective or late fusion schemes, modality-aware loss weighting, and visual alignment checks as effective mitigation strategies. These insights not only inform best practices for synthetic data utilization but also guide the design of robust fusion architectures tailored to the informational content of each modality. These strategies provide actionable guidance for applying synthetic datasets to real-world multimodal perception tasks across diverse domains.

Looking ahead, we aim to extend SegGen by integrating additional sensor modalities such as LiDAR and multispectral channels, exploring adaptive fusion mechanisms that dynamically gate modality contributions, and validating synthetic-to-real transfer, which helps enable applications such as environmental monitoring, precision agriculture, autonomous forest drones, and training generalizable multimodal perception models. By providing a flexible, scalable, and publicly available synthetic data generation pipeline, SegGen empowers researchers and practitioners to accelerate development of multimodal perception systems in under-served scenarios. 

## Figures and Tables

**Table 1:** Representative synthetic datasets for computer vision.

Dataset/Work	Domain	Modalities	Key Contribution
Playing for Data [15]	Gaming/Urban	RGB	Demonstrated video games as a source of semantic labels
Virtual KITTI 1/2 [16,17]	Driving	RGB, Depth, Flow	Synthetic driving benchmarks with pixel-level annotations
SYNTHIA [10]	Driving	RGB, Depth	Large-scale synthetic driving dataset
Sentinel-2 (Clabaut) [18]	Remote sensing	Multispectral	Synthetic Sentinel-2 segmentation
Unity Perception [19]	General	RGB, Depth, 3D	Toolkit for generating synthetic datasets with perfect labels

**Table 2:** Procedural and multimodal dataset contributions.

Dataset / Work	Domain	Modalities	Key Contribution
Nunes et al. [25]	Forestry	RGB, Depth	PCG-based synthetic forest environments
FinnWoodlands [26]	Forestry	RGB, Depth, LiDAR	Multimodal forestry dataset
M2fNet [9]	Forestry	RGB, Depth	Multimodal forestry dataset
Sen12MS [27]	Remote sensing	Multispectral	Land cover mapping dataset
SCaRL [11]	Driving	RGB, LiDAR, Radar	Game engine-based multimodal data
SDAC [28]	Driving	RGB, LiDAR, Radar	Anomaly/corner case detection

**Table 3:** Comparison of SPREAD and SegGen.

Dataset	Domain	Modalities	Automation	Focus
SPREAD	Forestry	RGB, Depth, Tree Params	Semi-automated	Tree segmentation and parameter estimation
SegGen (ours)	Remote sensing (forests, urban, land cover, etc.)	RGB, Depth, (extensible)	Fully automated	General multimodal semantic segmentation across diverse environments

**Table 4:** Pixel-level class distribution in the training split.

Class	Pixels (%)	Std. Across Images (%)
Ground	39.63	9.35
European Beech	31.50	17.22
Rocks	14.07	20.73
Norway Maple	9.40	6.53
Dead Wood	0.35	0.28
Black Alder	5.05	3.14

**Table 5:** Intersection-over-Union results on the benchmark Unreal Engine 5 Dataset. The dataset contains RGB and depth modalities. Bold values indicate the highest result in each column for each modality configuration.

Method	Modals	mIoU	Ground	Beech	Rocks	Maple	Dead	Alder
UNet	RGB	84.87	88.88	91.88	88.49	82.21	64.08	79.66
SegFormer	RGB	85.54	90.67	93.33	90.03	**85.59**	69.95	**83.70**
CMNeXt	RGB	82.60	88.90	91.85	88.81	82.22	64.17	79.65
FuseForm	RGB	**85.83**	**90.71**	**93.37**	**90.14**	85.50	**71.64**	83.62
UNet	RGB–Depth	81.99	88.75	91.86	88.28	81.95	62.22	78.89
SegFormer	RGB–Depth	83.87	89.77	92.92	88.97	84.30	65.68	81.61
CMNeXt	RGB–Depth	82.15	88.76	91.87	88.49	82.08	62.33	79.38
FuseForm	RGB–Depth	**85.03**	**90.43**	**93.30**	**89.67**	**85.20**	**68.67**	**82.90**

**Table 6:** Accuracy results on the benchmark Unreal Engine 5 Dataset. The dataset contains RGB and depth modalities. Bold values indicate the highest result in each column for each modality configuration.

Method	Modals	OA	Ground	Beech	Rocks	Maple	Dead	Alder
UNet	RGB	93.92	93.40	96.18	96.41	89.18	72.70	87.78
SegFormer	RGB	95.00	93.99	**96.89**	**97.41**	**93.32**	80.40	90.84
CMNeXt	RGB	90.39	92.99	96.37	96.47	89.25	77.86	89.38
FuseForm	RGB	**95.02**	**94.25**	96.84	97.01	91.76	**82.04**	**91.49**
UNet	RGB–Depth	93.83	92.94	96.54	96.59	88.68	70.69	87.95
SegFormer	RGB–Depth	94.51	93.13	97.08	**97.04**	**91.38**	**78.46**	**89.52**
CMNeXt	RGB–Depth	89.78	93.01	96.15	96.39	89.56	74.52	89.02
FuseForm	RGB–Depth	**94.88**	**94.08**	**97.17**	96.81	91.35	77.62	89.48

**Table 7:** Aggregate performance and impact of adding depth. Bold indicates the column best.

Method	mIoU			OA		
	RGB	RGB–D	Δ	RGB	RGB–D	Δ
UNet	84.87	81.99	−2.88	93.92	93.83	−0.09
SegFormer	85.54	83.87	−1.67	95.00	94.51	−0.49
CMNeXt	82.60	82.15	−0.45	90.39	89.78	−0.61
**FuseForm**	**85.83**	**85.03**	−0.80	**95.02**	**94.88**	−0.14

**Table 8:** Task-specific mechanisms by which an informatively flat depth map harms segmentation.

Failure Mode	Explanation and Typical Symptom
Semantic non-discriminativeness	Depth values overlap heavily across species; the optimiser down-weights RGB texture to minimise joint loss, eroding inter-species mIoU while OA (dominated by Ground) remains stable.
Gradient conflict	Texture-driven RGB gradients seek to separate foliage types; depth gradients encourage grouping all canopy pixels together. Without a conflict-resolution mechanism (e.g., PCGrad) the network converges to a compromise that is sub-optimal for both.
Capacity dilution	Early-fusion layers must now encode an additional, largely redundant channel, reducing effective capacity for fine texture cues—especially harmful to the under-represented Dead and Alder classes.

## Data Availability

Data supporting reported results can be found at https://github.com/Secure-and-Intelligent-Systems-Lab/SegGen (accessed on 23 August 2025).

## Abbreviations

The following abbreviations are used in this manuscript:

UE5	Unreal Engine 5
RGB	Optical Image data
ML	Machine Learning
LiDAR	Light Detection and Ranging
PCG	Procedural Content Generation
MiT	Mix-Transformer
IoU	Intersection-over-Union
OA	Overall Accuracy
CA	Class Accuracy
